# Data relating neurodevelopment of exclusively breastfed children of urban mothers and pre- and post-natal mercury exposure

**DOI:** 10.1016/j.dib.2019.104283

**Published:** 2019-08-07

**Authors:** Rejane Correa Marques, José G. Dórea, José Vicente Elias Bernardi, Wanderley Rodrigues Bastos, Olaf Malm

**Affiliations:** a Universidade Federal do Rio de Janeiro, Brazil; b Universidade de Brasília, Brazil; c Universidade Federal de Rondonia, Brazil

**Keywords:** Child development, Fish, Mercury exposure, Hair mercury

## Abstract

A cohort of 100 exclusively breastfed children was formed to study association between neurodevelopment and Hg exposure [1]. Detailed questionnaires were administered by trained interviewers to the mothers, and measurements of early child development were collected at 6, 36 and 60 months by trained nurses and psychologists using the Gesell Developmental Schedules. The association between prenatal and postnatal organic Hg (methyl-Hg from fish consumption and ethyl-Hg from Thimerosal-containing vaccine) exposures with Gesell scores of the four domains [Adaptive ability, Language development, Motor abilities (gross motor), and Personal-social ability] were studied in preschool children.

**Table:** Specifications Table

Subject area	Human Biology
More specific subject area	Child Development and Growth.
Type of data	Text and Tables
How data was acquired	Longitudinal cohort study, questionnaire data (supplemental file); biological assessment
Data format	Edited; raw data are provided as a supplemental file
Experimental factors	Maternal questionnaires (supplemental file), Gesell Developmental Schedules, hair assays for total mercury, and immunization cards for ethyl-Hg in Thimerosal-containing vaccines.
Experimental features	Children's Gesell scores at 6 months, 3 and 5 years in association with hair-Hg concentrations.
Data source location	Universidade Federal do Rio de Janeiro, Rio de Janeiro, Brazil.
Data accessibility	Data are within this article.
Related research article	[1] Marques R.C., Dorea J.G., Bastos W.R., Malm O. Changes in children hair-mercury concentrations during the first five years: maternal, environmental and iatrogenic modifying factors. Regul Toxicol Pharmacol, v. 49, p. 17–24, 2007a. https://doi.org/10.1016/j.yrtph.2007.05.001

**Table:** 

**Value of the Data** • This dataset is useful because it covers a geographically defined population (Porto Velho, RO, Brazil) with habitual high levels of fish consumption. • The data benefit understanding neurodevelopment in exclusively breastfed infants in association with exposure to environmental fish-mercury. • The data levels of family fish consumption (hair-Hg) and socioeconomic factors useful to public health and environmental scientists studying child neurodevelopment. • The additional value of the data is related to documenting the changing environment and demographics of Western Amazon.

## 1 Data

We describe data on child development, taking into consideration maternal fish consumption during pregnancy and lactation and infant exposure to ethyl-Hg in Thimerosal-containing vaccines. Dataset were summarized in Table 1, Table 2 (raw data are provided as a supplemental file). The dataset was designed to answer questions related to organic Hg exposure (from different sources) during prenatal and postnatal Hg exposures of breastfed infants and to evaluate children's growth and neurodevelopment. Pregnant mothers attending pre-natal clinics of three hospitals in Porto Velho were introduced and invited to participate voluntarily in the study. At hospital delivery and during regular visits (6, 36, and 60 months) we applied and/or updated questionnaires (see supplemental file), took anthropometric measurements and hair samples from mothers and children; at these visits, trained nurses applied neurodevelopment tests and made an evaluation of children's health [1], [2]. The Ethics Committee for Studies in Humans of the Federal University of Rondonia approved the study.

**Table 1:** Publications using the questionnaire data, measurements of child growth and Gesell Scores.

Authors	Outcomes	Environment	Results
Marques et al., 2007a [1]	0, 6, 36 and 60 months	Hg exposure (injected EtHg and hair-Hg)	The distribution of hair-Hg in mothers and infants followed different patterns. Extended breastfeeding (6–24 months) was not significantly associated with changes in hair-Hg of mothers or infants.
Marques et al., 2007b [2]	Birth and 6 months	Neuro-motor development	Neurodevelopment is not associated with fish consumption (hair-Hg) but could be attributed to health inequalities and social deprivation.
Marques et al., 2007c [7]	6 months	Hg exposure (injected EtHg and hair-Hg) and exclusively breastfeeding	The dose and parenteral exposure mode of TCV (ethyl-Hg) seemed to influence the relative increase in hair-Hg concentrations of six-month-old breastfed infants.
Marques et al., 2007d [8]	6 months	Gesell Developmental Schedules and Hg exposure (injected EtHg and hair-Hg)	Differences in early exposure to TCV (ethyl-Hg) cannot predict neurodevelopment delays in six-month-old breastfed infants.
Marques et al., 2009 [9]	0, 6, 36 and 60 months	Infant hair-Hg and Gesell Developmental Schedules	The observed developmental delays associated with TCV (ethyl-Hg) in six-month-old breastfed infants were overcome by 36 months of age.
Marques et al., 2008a [10]	0, 6, 36 and 60 months	Maternal fish consumption during pregnancy and lactation	Fish consumption (hair-Hg) had no statistically significant impact on children's anthropometry. However, breastfeeding duration was significantly correlated with attained Z-scores for weight-for-height and weight-for-age.
Marques et al., 2008b [11]	6 months	Gesell Developmental Schedules	Principal Component Analysis could discriminate variability of early TCV (ethyl-Hg) exposure and Gesell Developmental Schedules outcomes associated with variables that included pre- and post-natal environmental Hg exposure.

**Table 2:** Infant anthropometry, neurodevelopment schedules and markers of Hg exposure during the first 5 years.

	Mean ± SD	Median	Min	Max
***Birth***				
Weight, kg	3.23±0.42	3.20	2.20	4.37
Length, cm	49.77±2.43	50.00	35.00	55.00
Head circumference, cm	33.88±2.29	34.00	30.00	48.00
*Tissue Hg concentrations*				
Mother's Blood Hg, μg/L	0.98±1.51	0.55	0.01	9.97
Placenta, μg.g^−1^	10.75±9.82	8.11	0.37	56.28
Umbilical cord, ng.g^−1^	10.54±9.93	7.45	0.12	43.74
Mother's Hair Hg, μg.g^−1^	7.36±8.73	5.41	0.39	62.43
Infant's hair Hg, μg,g^−1^	2.44±3.04	1.60	0.05	19.65
Fish Meals (week)	1.97±2.81	1	0	14
***6 Months***				
Weight, kg	7.01±0.46	7.00	6.11	8.50
Length, cm	67.60±2.59	68.00	61.00	73.00
Head circumference, cm	42.58±1.08	43.00	40.00	45.00
*Hg concentrations*				
Mother's Hair Hg, μg.g-^1^	3.19±3.85	2.17	0.10	22.87
Infant's hair Hg, μg.g-^1^	3.84±5.55	1.82	0.02	32.95
TCV (EtHg), μg	181,40±39,25	175.00	150.00	325.00
*Neurodevelopment schedules*				
Motor	74.82±31.95	85.00	0.00	100.00
Language	67.87±36.24	85.00	0.00	100.00
Adaptive	82.01±19.62	85.00	0.00	100.00
Personal-social	86.10±10.21	85.00	70.00	100.00
Total DQ	77.70±22.25	84.00	18.00	100.00
***3 years***				
Weight, kg	15.30±1.85	15.40	11.00	19.50
Height, cm	96.30±3.45	97.00	88.00	103.00
Head circumference	50.27±1.22	50.00	48.00	53.00
*Tissue Hg concentrations*				
Mother's Hair Hg, μg.g^−1^	2.75±3.20	1.39	0.10	15.60
Infant's hair Hg, μg.g^−1^	2.75±3.20	1.39	0.10	15.60
*Neurodevelopment schedules*				
Motor	83.73±15.23	85.50	35.00	100.00
Language	80.12±18.27	85.00	30.00	100.00
Adaptive	86.70±10.65	88.00	55.00	100.00
Personal-social	88.09±11.06	90.00	45.00	100.00
Total DQ	84.66±11.81	85.25	54.50	100.00
***5 years***				
Weight, kg	19.19±1.39	19.50	16.00	22.00
Height, cm	106.79±3.06	107.00	100.00	115.00
*Tissue Hg concentrations*				
Mother's Hair Hg, μg.g^−1^	2.64±3.53	1.35	0.10	15.67
Infant's hair Hg, μg.g^−1^	2.6±3.5	1.35	0.10	20.2
*Neurodevelopment schedules*				
Motor	82.62±13.01	80.00	50.00	100.00
Language	79.21±16.56	80.00	40.00	100.00
Adaptive	83.96±12.04	85.00	50.00	100.00
Personal-social	86.33±11.37	90.00	50.00	100.00
Total DQ	83.03±12.08	83.88	61.25	100.00
Length of lactation, m	14.55±3.65	14.00	9.00	26.00

TCV = Thimerosal-containing vaccine, DQ = development quotient.

* All 82 infants had a full immunization schedule during the first six months; the fourth dose of DTP was taken after the first year only by 25.5%. The Hib and influenza vaccines at six months were taken at the recommended age by the specified percentage of the children; the ages of immunization after 6 months are approximations based on current recommendations.

^#^Hp-B: Hepatitis B (0.01%Thimerosal/dose, Korea Green Cross Corporation, Kiheung-Eup Yougin-Goon Kiyunggi-Do, Korea; Euvax B injectable, 0.01%Thimerosal [LG Life Sciences, Jeonbuk-do, Korea]); DTP (Serum Institute of India Ltd; Vacina Tríplice, 0.01%Thimerosal/dose [Instituto Butanta, São Paulo, Brazil]); Hib: Haemophilus influenzae type b (HibTITER®, 0.01% Thimerosal/dose, Lederle-Praxis); Flu: influenza (VAXIGRIP 0.01%Thimerosal/dose, Pasteur Mérieux Connaught, Sao Paulo, Brazil).

Children that were immunized against *Haemophilus influenzae* type b (Hib) within the first year (2 doses) received the booster dose 6–12 months after the last dose; otherwise, they received only one dose in the second year of life. The recommendation for the Anti-flu vaccine is to be taken between the ages of six to 35 months in two doses (one month apart) of 0.25mL and one single dose (0.5mL) the following year; although the vaccines were taken the ages given represent approximations of the recommended date.

## 2 Experimental design, materials, and methods

### 2.1 Participants

This study began in the year 2000, aiming to assess the impact of fish consumption and Hg exposure on children growth and neurodevelopment. The participating mothers resided in the capital city of Porto Velho (state of Rondonia, Western Amazonia). The selected pregnant mothers were in good health, without apparent illnesses or complaints, and were willing to breast feed (exclusively up to six months of age); they also manifested interest in continuing post-natal visits to the pediatric clinic that included the recommended immunization scheme. One hundred mothers (aged 15 to 45 years) took part in the study. For each mother, at the time of the first interview, a complete clinical evaluation was extracted from medical records; a questionnaire applied by trained nurses collected data on socioeconomic and educational status, food habits (fish consumption), and breastfeeding practices. At six months of age, only 86 mother-infant pairs reported for the first clinical and neurobehavioral examination: five mothers moved away, five did not report and/or could not be found, four babies died within the first month, two mothers developed gestational diabetes, and two others developed pre-eclamptic toxemia. Therefore, we ended up with complete data from 82 mother-infant pairs [2]. During the study, nurses assisted the mothers with pre- and postnatal care to guarantee full breastfeeding support.

### 2.2 Questionnaire assessments

In the follow-up programmed visits (6, 36, and 60 months), a trained interviewer applied questionnaires to collect and/or update information on socioeconomics, family fish consumption, breastfeeding practices, age of walking and age of talking. Socioeconomic information included number of persons per home, family income and related maternal educational level variables.

### 2.3 Anthropometric measurements

Hospital medical records provided clinical (Apgar scores) and anthropometric (weight, length, head circumference) data on the newborns, which included vitality, perinatal reflexes, maturity, and congenital malformations. At ages of 6, 36 and 60 months, trained nurses took anthropometric measurements (weight and height) of mothers and infants with standard procedures. Newborns and six month-old infants were measured in recumbent position and older children (36 and 60 months) had standing height taken barefoot to the nearest 0.1 cm with a stadiometer; the children, dressed in underwear, were weighed with an electronic scale to the nearest 0.1 kg. All children's measurements had Z-scores for attained weight-for-age, length-for-age, BMI-for-age and weight-for-length based on the WHO Child Growth Standards [3].

### 2.4 Neurodevelopment measurements

Trained professionals applied the Gesell Developmental Schedules to assess neurodevelopment [4], [5]. The Gesell tests include voluntary, spontaneous or learned reactions and reflexes; the tests evaluate: locomotion and coordination, postural reactions, constructive ability, hand pressure, visible and audible communication, and individual reactions regarding people and stimulations. The outcomes are compared to those expected for the appropriate developmental stage corresponding to the respective age. These results (in percentages) are expressed as developmental quotients (DQ) for Language Development, Adaptive Behavior, Gross Motor Skills and Personal/Social Behaviors.

### 2.5 Hair collection and mercury determination

At delivery, while mothers were in the maternity wards, samples of cord blood (4.5 mL), placenta (three different aliquots) and, umbilical cord (5–10 g) were collected and properly stored (acid-clean glassware) under refrigeration, taken to the analytical laboratory and kept frozen (−20 °C) until analysis [2]. Hair strands (from mothers and newborns) were cut from the occipital region, the root end stapled on a paper sheet, and placed in properly tagged plastic bags. Repeated head-hair samples were taken during the follow-up visits (6, 36 and 60 months). Hg determination was done after sample preparation according to routine procedure used at the Universidade Federal do Rio de Janeiro [2], [6]; after comminution with stainless steel scissors, the hair samples were weighed and digested before analysis. After washing with EDTA (0.01%) hair samples were dried (50 °C) and an aliquot was weighed before acid digestion (5 mL of HNO_3_:H2SO_4_ (1:1) and 4 mL of 5% KMnO_4_) in a digestion block for 40 min. (80 °C). Sample total Hg was determined by cold vapor atomic absorption spectrometry (CV-AAS) coupled with a flow injection system (FIMS, Perkin-Elmer—FIMS 400, Ueberlingen, Germany). Precision and accuracy of Hg determinations were checked with internal standards, triplicate analyses, and certified reference materials (IAEA-085 and 086, Vienna, Austria). The tested recoveries were of 92%, and the limit of detection was determined at <0.01 μg/g. No hair-Hg concentration was found below the limit. All glassware used in the hair analysis was previously washed clean, and rinsed with both EDTA (5%) and double-distilled water. The cleaning process was continued after overnight resting in acid solution (5% HNO_3_)_,_ rinsed again in double-distilled and deionized water; the material was then dried at 100 °C for 12 h.

### 2.6 Exposure to EtHg from Thimerosal-containing vaccines (TCVs)

Immunization with TCVs followed the Brazilian vaccination program: Hepatitis B (taken either before hospital discharge or a few days later) and DTP at 30, 60, 120 and 160 days at a state-run clinic where vaccines are distributed free. All infants up to 6 months of age were fully immunized, as recommended by the Brazilian immunization scheme. According to information provided by the vaccine manufacturers, the pediatric TCVs (hepatitis-B - Korea Green Cross Corporation, Kiheung-Eup Yougin-Goon Kiyunggi-Do, Korea; Euvax B injectable - LG Life Sciences, Jeonbuk-Do, Korea; diphtheria, tetanus and pertussis (DTP) - Triple Antigen, Serum Institute of India Ltd, India; Vacina Tríplice, Instituto Butanta, São Paulo, Brazil; Haemophilus influenzae type b (Hib) - HibTITER®,Lederle-Praxis; influenza (Flu) - VAXIGRIP, Pasteur Merieux Connaught, Sao Paulo, Brazil) were preserved with 0.01% Thimerosal (which metabolizes into ethyl-Hg); this dose corresponds to 25 μg Hg/0.5 mL. The immunization schedule with TCVs and ethyl-Hg body load was estimated based on the national immunization scheme adopted by the Ministry of Health of Brazil. Only 20 infants received a fourth dose of DTP between the ages of 9 and 12 months [7], [8].

### 2.7 Publications

Publications using the questionnaire data, neurodevelopment outcomes and infant growth are shown in Table 1. Positive associations were found between neurodevelopment and maternal education [2] and length of lactation [9]. Hair-Hg concentrations (marker of fish consumption) were negative and significantly correlated with Gesell scores at 6 months and 60 months [9]. No statistically significant association was observed between maternal/child hair-Hg and length of lactation [1]. In regard to TCV-EtHg, the time of exposure showed no significant association with neurodevelopment delay [8], but it seems that the mode of Hg exposure (parenteral EtHg) and dose influenced the relative increase in hair-Hg of six-month-old breast-fed infants [7]. Additionally, family fish consumption (as hair-Hg) showed no impact on linear growth and development of children [1], [10].
