# Supplementary material for: Data relating neurodevelopment of exclusively breastfed children of urban mothers and pre- and post-natal mercury exposure
Source: Data Brief. 2019 Aug 7;25:104283. doi: 10.1016/j.dib.2019.104283 (PMC6706763; doi:10.1016/j.dib.2019.104283)
Supplement: Multimedia component 1 [file mmc1.doc]

**Questionnaire to mothers with newborn**

**UNIVERSIDADE FEDERAL DE RONDÔNIA – UNIR**

**UNIVERSIDADE DE BRASÍLIA – UnB**

**UNIVERSIDADE FEDERAL DO RIO DE JANEIRO – UFRJ**

**Project: Mercury exposure in children from Porto Velho, Rondonia, Brazil**

**HOUSING AND FAMILY LIVING CONDITIONS:**

How many people in the household:_________

Home ownership: ( ) owned ( )rented ( ) Relatives` house ( ) home loaned by others

Type of building: ( ) wood ( ) masonry ( ) “taipa” ( ) in flooded ground

Presence of insects and rodents: ( )no ( )yes, type:.................................................................

Electricity: ( )yes ( )no

Sanitation (Feces disposal): “( )fossa seca”/septic tank “( )fossa negra”/open-sewage pit ( )dug-out pit ( )above-ground defecation ( )river

Water supply: ( )plumming ( )river ( )well ( )public stand-pipe ( )other:...........

**MOTHER**

Name:____________________________________________________________________

Birth Date: ___/___/___ Age (years):____

Race classification: Black ( ) Mixed race ( ) Indigenous ( ) White ( )

Address:_____________________________________ Phone:_____________________

Mother Schooling (years)_______; Father's schooling:___________years

Income: R$____________

Mining work: no( ); yes( ), from______to ______

How long living at the current address:______years

Meals per day:___________

Fish consumption: ( )no, ( )yes meals per week:___________________

Kind of fish:___________________ Source:__________________

Use of charcoal for cooking: ( )no ( )yes frequency:__________ Indoor stove ( )yes ( )no

Exposure to pesticides or similar substances: ( )no ( )yes Type:_________________________

Use of recreational drugs: ( )no ( )yes Type:_________________________ period:_______

Use of medication: ( )no ( )yes Type:_________________________ period:______

Use of alcohol: ( )no ( )yes frequency:________ Type of drink_________ quantity/day:_______

**Obstetrical information**

Prenatal: yes ( ) no ( ) number of visits:

Mode of delivery: ( ) vaginal ( )forceps ( ) Cesarean section

Birth environment: ( ) hospital ( ) home

Gestational Age:______weeks

Number of Children: _______

**Newborn data**

Sex: ( ) Female ( ) Male

Apgar Score: 1^st^ min _______ 5^th^ min________

Anthropometry: weight:________g length:______cm head circumference:____cm

Birth: ( ) term ( ) pre-term ( ) Post-term

Nutritional status at term: ( ) Appropriate for gestational age/AGA ( ) Small for gestational age/SGA ( ) Large for gestational age/LGA

Congenital anomalies: ( )no ( ) yes: ______________

Interviewer:........................................................................

**Questionnaire (6 months, 3-5year old children)**

**UNIVERSIDADE FEDERAL DE RONDÔNIA – UNIR**

**UNIVERSIDADE DE BRASÍLIA – UnB**

**UNIVERSIDADE FEDERAL DO RIO DE JANEIRO – UFRJ**

**Project: Mercury exposure in children from Porto Velho, Rondonia, Brazil**

Name: _________________________________________________________________

Birth date: ___/___/____ Age:_________ Sex: ( )Male ( )Female

Race classification: Black ( ) Mixed race ( ) Indigenous ( ) White ( )

Place of birth: ____________________________________________________________________

Current address:___________________________________________________________________

Mother's name: ______________________________________ occupation: ___________________

Father's name: _______________________________________ occupation:___________________

Family income: __________________ Phone:____________

Number of siblings____________________ Relationship to interviewee:___________________

Child attending daycare facility/school: ( )yes ( )no

**FEEDING HISTORY**

Breastfeeding (months):_______ meals/ day:_____ most frequent foods:___________________

Fish Consumption: ( )yes ( )no, fish meal per week:___________________

**HEALTH**

**Vaccinaton History:**

Child health record book: ( )yes ( )not vaccinated

| Vaccines | Dates | | | | | |
| --- | --- | --- | --- | --- | --- | --- |
|  |  |  |  |  |  |  |
|  |  |  |  |  |  |  |
|  |  |  |  |  |  |  |
|  |  |  |  |  |  |  |
|  |  |  |  |  |  |  |
|  |  |  |  |  |  |  |
|  |  |  |  |  |  |  |
|  |  |  |  |  |  |  |
|  |  |  |  |  |  |  |
|  |  |  |  |  |  |  |
|  |  |  |  |  |  |  |
|  |  |  |  |  |  |  |

Adverse effects following vaccination ( )no; ( ) yes, prescribed medicine: ( )no ( ) yes_____

Diseases ____________________________________________________________________

**Anthropometry**

Weight:___________Height:_______ Head circumference:_______

Other findings:__________________________________________________________

**NEURODEVELOPMENT**

***Infant milestones*** (in months)

Age of Walking:________ Age of Talking:_______________

Diagnostic impression:___________________________________________________

**Gesell Schedules**

| **Motor** |  |  |
| --- | --- | --- |
| **Adaptive** |  |  |
| **Language** |  |  |
| **Personal-Social** |  |  |

Interviewer:_______________________________________
